# Fumarate Hydratase-Deficient Uterine Leiomyomas: Germline Genetic Findings and Clinical Follow-Up in a Single-Center Cohort

**DOI:** 10.3390/diagnostics16182985

**Published:** 2026-09-15

**Authors:** Nisan Helin Donmez, Elif Yilmaz Gulec, Hanne Bulat Cim, Begüm Sinem Kezer Cingoz, Melis Altug Inan, Emine Bolukbasi Karapinar, Ergul Demircivi, Canan Satir Ozel, Abdulkadir Turgut

**Affiliations:** 1 Department of Obstetrics and Gynecology, Prof. Dr. Süleyman Yalçın City Hospital, Istanbul 34722, Türkiyemelaltug@gmail.com (M.A.I.); 2 Department of Medical Genetics, Faculty of Medicine, Istanbul Medeniyet University, Istanbul 34730, Türkiye; 3 Department of Medical Genetics, Prof. Dr. Süleyman Yalçın City Hospital, Istanbul 34722, Türkiye; 4 Department of Obstetrics and Gynecology, Faculty of Medicine, Istanbul Medeniyet University, Istanbul 34730, Türkiye

**Keywords:** FH-deficient leiomyoma, *FH*, hereditary leiomyomatosis and renal cell cancer, FH tumor predisposition syndrome, uterine leiomyoma

## Abstract

**Background**: Fumarate hydratase (FH)-deficient uterine leiomyomas are rare tumors that may be associated with germline *FH* variants and FH tumor predisposition syndrome. This study aimed to evaluate the clinical characteristics, germline genetic findings, and follow-up outcomes of patients with FH-deficient uterine leiomyomas, and to determine the frequency of germline pathogenic/likely pathogenic (P/LP) *FH* variants. **Methods**: This ambispective single-center cohort study included 19 patients diagnosed with FH-deficient uterine leiomyomas at a center between February 2021 and February 2026. Clinical characteristics, surgical data, FH and S-(2-succino) cysteine (2SC) immunohistochemical findings, germline *FH* analyses, renal imaging, dermatologic evaluation, and follow-up outcomes were reviewed. **Results**: Mean age was 44.9 ± 7.2 years, and the median largest leiomyoma diameter was 6.5 cm. Loss of FH expression was observed in 17/19 cases, while diffuse 2SC positivity was present in all cases. Among 17 patients who underwent germline testing, two (11.8%) harbored P/LP *FH* variants, [c.434C>G, p.(Ser145Ter) and c.1256C>G, p.(Ser419Ter)], and one (5.9%) had a variant of uncertain significance (VUS) [c.1090G>A, p.(Gly364Arg)]. Dermatologic evaluation was completed, with no cutaneous leiomyomas identified; no renal malignancy was detected on imaging in the two P/LP variant carriers. Among nine patients who underwent myomectomy, three developed recurrence; the patient carrying the likely pathogenic variant conceived after myomectomy. **Conclusions**: Identification of an FH-deficient uterine leiomyoma on pathologic examination is an important finding that should prompt evaluation for germline FH variants. These patients require integrated genetic, renal, dermatologic, and gynecologic follow-up. Obstetricians and gynecologists have an important role in initiating genetic evaluation and long-term surveillance after surgery.

## 1. Introduction

Uterine leiomyomas are the most common benign uterine smooth muscle tumors in reproductive-age women [1]. Approximately 0.4–1.6% are fumarate hydratase (FH)-deficient, a distinct molecular and histopathologic subtype characterized by loss of FH function [2].

The fumarate hydratase (*FH*) gene, located on chromosome 1q43, is a highly conserved tumor suppressor encoding an enzyme involved in the mitochondrial tricarboxylic acid cycle and nuclear DNA double-strand break repair [3]. Loss of FH function causes fumarate accumulation, which promotes protein succination, epigenetic dysregulation, oxidative stress, impaired DNA damage response, and stabilization of hypoxia-inducible factor alpha (HIF-α) through the inhibition of prolyl hydroxylases. These alterations support cellular proliferation, angiogenesis, metabolic reprogramming, and tumor progression.

In uterine leiomyomas, FH deficiency may arise from somatic alterations or from germline pathogenic *FH* variants associated with hereditary leiomyomatosis and renal cell carcinoma syndrome [4,5]. Beyond its implications for hereditary cancer predisposition, FH deficiency also has important diagnostic relevance. FH-deficient uterine leiomyomas may exhibit marked nuclear atypia and other atypical histomorphologic features that overlap with those of smooth muscle tumors of uncertain malignant potential (STUMP) and uterine leiomyosarcoma. Although FH deficiency has also been reported in a small subset of uterine leiomyosarcomas, there is currently no direct evidence that FH-deficient leiomyomas carry an increased risk of malignant transformation to leiomyosarcoma, and the biological significance of this association remains uncertain [6]. Therefore, careful interpretation of FH deficiency in conjunction with clinical, histomorphologic, and immunohistochemical findings is important both for identifying patients who may warrant genetic counseling and renal malignancy risk assessment and for ensuring accurate classification of uterine smooth muscle tumors while minimizing potential diagnostic misclassification.

Hereditary leiomyomatosis and renal cell cancer (HLRCC) syndrome is an autosomal dominant cancer predisposition syndrome caused by heterozygous germline pathogenic *FH* variants and characterized by cutaneous and uterine leiomyomas and FH-deficient renal cell carcinoma, which often exhibits aggressive biologic behavior [7,8,9]. Uterine leiomyomas are among the most common manifestations in affected women, typically arise in the third decade, present with abnormal uterine bleeding and pelvic pain, are frequently multiple, may grow rapidly, and can precede renal malignancy [10]. Cutaneous leiomyomas predominantly involve the trunk and extremities and may increase in number and size with age, whereas renal tumors are usually solitary and unilateral and may metastasize even when the primary tumor is small [11]. Accordingly, identification of FH deficiency in a uterine leiomyoma should prompt germline genetic evaluation, renal malignancy screening, and assessment of at-risk relatives, as these tumors may represent an early marker of hereditary cancer predisposition [12].

FH-deficient uterine leiomyomas were initially described mainly in case reports and small series; however, larger clinicopathologic cohorts have been published in recent years [13]. Contemporary studies have more clearly characterized the association between FH deficiency and germline pathogenic *FH* variants, the clinical significance of these findings, and the expanding genetic spectrum associated with the identification of novel *FH* variants [14,15]. Nevertheless, there remains a need for comprehensive studies integrating clinical, histopathologic, immunohistochemical, and genetic findings. A recently published retrospective Turkish series of 10 patients described the clinical, familial, and immunohistochemical characteristics of FH-deficient uterine leiomyomas, together with renal and dermatologic evaluations; however, systematic germline *FH* analysis was not performed [16]. Therefore, comprehensive data incorporating the systematic germline evaluation of hereditary *FH* predisposition remain limited in the Turkish population. The present study aims to address this gap by providing an integrated assessment of clinical, histopathologic, immunohistochemical, germline genetic, renal, dermatologic, and follow-up findings from a Turkish cohort, thereby contributing complementary data to the predominantly Western literature.

We aimed to evaluate the clinical characteristics, germline *FH* findings, and follow-up outcomes of 19 patients with FH-deficient uterine leiomyoma managed at a tertiary center over five years. We also assessed the frequency of germline pathogenic/likely pathogenic (P/LP) *FH* variants and characterized renal, dermatologic, fertility-related, and recurrence outcomes. This integrated approach was intended to clarify the clinical relevance of FH-deficient uterine leiomyoma for germline genetic evaluation, surveillance considerations, and clinical follow-up.

## 2. Materials and Methods

### 2.1. Study Design, Population, and Data Collection

This ambispective, single-center cohort study consisted of a retrospective review of existing clinical, pathologic, imaging, and germline genetic data, supplemented by a prospective data-completion and follow-up component. The study included 19 patients diagnosed with FH-deficient uterine leiomyomas based on the histopathologic and immunohistochemical evaluation of myomectomy or hysterectomy specimens at a tertiary referral center between February 2021 and February 2026.

In the retrospective component of the study, age, body mass index (BMI), gravidity, parity, presenting symptoms (abnormal uterine bleeding, pelvic pain, or asymptomatic presentation), family history of uterine leiomyoma or renal cell carcinoma, preoperative clinical and imaging findings (pelvic examination, Doppler ultrasonography, and magnetic resonance imaging when available), largest tumor diameter, tumor number, surgical procedure, histopathologic and immunohistochemical findings, and all available germline FH analysis results obtained from peripheral blood were extracted from electronic medical records. In addition, all available renal imaging records were reviewed retrospectively; imaging performed within the preceding 12 months was considered a current renal assessment. Postoperative gynecologic follow-up records and available dermatologic assessments for cutaneous leiomyomas were also retrospectively reviewed and included in the study. Data regarding leiomyoma recurrence were obtained from clinical assessments and ultrasonographies performed during annual gynecologic follow-up.

During the prospective data-completion and follow-up phase conducted in 2026, patients were re-contacted and their family histories of uterine leiomyoma and renal cancer were reassessed. Patients who had not undergone gynecologic follow-up for leiomyoma assessment within the preceding 12 months were invited for clinical and ultrasonographic evaluation. In patients with germline P/LP *FH* variants, renal and dermatologic evaluations performed within the preceding 12 months were reviewed, and renal imaging and dermatologic evaluations were arranged when these assessments were incomplete. In addition, patients carrying P/LP variants were referred to the Medical Genetics unit for assessment of familial risk and cascade genetic evaluation, whereas the patient with a VUS was referred for assessment of the clinical significance of the genetic finding and for appropriate genetic counseling.

### 2.2. Immunohistochemical Evaluation

Immunohistochemical findings were retrospectively obtained from the original pathology reports. FH and S-(2-succino)cysteine (2SC) immunostaining were performed as part of the routine diagnostic work-up in uterine leiomyomas showing morphologic features suggestive of FH deficiency. Results of additional markers, including desmin, h-caldesmon, Ki-67, and p53, were recorded when available.

No additional immunohistochemical staining or central pathology review was performed for this study. FH expression and 2SC staining patterns were extracted directly from the original pathology reports; FH was recorded as lost or retained, whereas 2SC was reported as diffusely positive in all cases. For cases with retained FH expression, the original pathology reports did not further characterize the staining pattern as weak, focal, mosaic, or partial; therefore, no additional subclassification of retained FH expression was possible.

### 2.3. Genetic Analysis

Genomic deoxyribonucleic acid (DNA) was isolated from peripheral-blood samples. Depending on the clinical indication, germline genetic testing was performed using either a hereditary cancer multigene panel including *FH* or targeted single-gene *FH* testing. DNA libraries were prepared using the Nextera Illumina Exome Oligo Kit (Illumina, San Diego, CA, USA) and sequenced on an Illumina NovaSeq platform (Illumina, San Diego, CA, USA) using Illumina V2 chemistry and the SOPHiA DDM™ Hereditary Cancer Solution (SOPHiA GENETICS, Rolle, Switzerland). A minimum sequencing depth of 100× was achieved.

Raw sequencing data were analyzed using the SOPHiA DDM^®^ (version 7.23.0.1)platform. Sequence alignment to the human reference genome hg38 and variant calling were performed using the PEPPER^®^ (release r0.8) algorithm, and variant annotation was conducted using MOKA^®^. The next-generation sequencing (NGS)-based bioinformatic pipeline included both single-nucleotide variant (SNV) analysis and copy-number variant (CNV) analysis in all NGS-tested patients. CNV analysis was performed using MUSKAT^®^. For each variant, the predicted effect on the protein sequence, allele frequency in population databases (1000 Genomes, Exome Sequencing Project [ESP], Exome Aggregation Consortium [ExAC], and Genome Aggregation Database [gnomAD]), and predicted functional impact using in silico tools (SIFT and PolyPhen) were assessed. Thus, large deletions and duplications were assessed in all NGS-tested patients, including those in whom no sequence variant was identified. No pathogenic or likely pathogenic CNV involving *FH* was detected. The identified germline variants were additionally reviewed using Integrative Genomics Viewer (IGV, version 2.6.3). Because the variants showed high sequencing depth, variant allele fractions compatible with heterozygous germline variants, and consistent bidirectional read support, additional confirmation by Sanger sequencing was not considered necessary. No findings suggestive of sequencing artifacts were observed on IGV review.

Variants were classified according to the American College of Medical Genetics and Genomics/Association for Molecular Pathology (ACMG/AMP) criteria and recommendations of the Clinical Genome Resource (ClinGen) Sequence Variant Interpretation Working Group, with ClinVar and the current literature used as supporting reference sources.

Because somatic *FH* analysis of tumor tissue was not performed, patients without a germline pathogenic/likely pathogenic *FH* variant were not classified as having sporadic disease. Germline findings were therefore reported as P/LP variants, variants of uncertain significance (VUS), or no P/LP/VUS detected.

### 2.4. Renal and Cutaneous Evaluation

Renal imaging findings were obtained from the available electronic medical records, and results of ultrasonography, computed tomography, and magnetic resonance imaging were recorded. Patients with germline P/LP *FH* variants were advised to undergo annual renal magnetic resonance imaging and periodic dermatologic surveillance. During the prospective data-completion phase, patients without renal imaging or dermatologic evaluation within the preceding 12 months were referred to the Radiology and Dermatology departments. Patients without a renal assessment within the preceding 12 months were classified as “not completed”.

### 2.5. Study Outcomes

The primary outcome was the frequency of germline pathogenic/likely pathogenic (P/LP) *FH* variants among patients with FH-deficient uterine leiomyomas. Variants of uncertain significance (VUS) were reported separately.

Secondary outcomes included demographic, clinical, and pathological characteristics; personal and family history related to FH tumor predisposition syndrome; renal imaging findings; cutaneous findings; fertility-related outcomes; and clinical follow-up findings.

### 2.6. Statistical Analyses

Statistical analyses were performed using jamovi software (version 2.6.45). The distribution of continuous variables was assessed using the Shapiro–Wilk test and visually examined using histograms and quantile–quantile (Q–Q) plots. Normally distributed continuous variables are presented as mean ± standard deviation, whereas non-normally distributed continuous variables are presented as median and interquartile range (IQR). Categorical variables are presented as n (%). For variables with missing observations, analyses were performed using the available data, and the number of patients included in each analysis was specified. No imputation was performed for missing data. Because the study was descriptive and no prespecified comparison groups were defined, no inferential statistical analyses or hypothesis tests were conducted.

Generative artificial intelligence was used solely to assist with English language translation and linguistic refinement of the manuscript. It was not used for study design, data collection, data analysis, or interpretation of the results.

## 3. Results

A total of 19 patients diagnosed with FH-deficient uterine leiomyoma over the five-year study period were included in the study.

### 3.1. Demographic, Clinical, and Surgical Findings

The mean age was 44.9 ± 7.2 years (range, 34–60); nine patients (47.4%) were premenopausal and 10 (52.6%) postmenopausal. Mean body mass index was 28.6 ± 6.5 kg/m^2^. Among 14 patients with available data, the median age at first leiomyoma diagnosis was 35.5 years (IQR, 30.3–40.0), and five patients (26.3%) were nulligravida.

Abnormal uterine bleeding was the most common presentation, occurring alone in eight patients (42.1%) and with pelvic pain in three (15.8%); four patients (21.1%) had pelvic pain alone and four (21.1%) were asymptomatic. A family history of uterine leiomyoma was reported in 11 patients (57.9%) and renal cancer in one (5.3%). Three patients (15.8%) had undergone previous leiomyoma surgery.

The median largest leiomyoma diameter was 6.5 cm (IQR, 4.5–8.0; range, 1.5–15.0). Ten patients (52.6%) underwent hysterectomy and nine (47.4%) abdominal myomectomy. Median postoperative follow-up was 30 months (IQR, 16–37). Demographic, clinical, and surgical characteristics are summarized in Table 1.

### 3.2. Histopathologic and Immunohistochemical Findings

The largest leiomyoma diameter ranged from 1.5 to 15 cm; 11 patients had single leiomyomas and eight had multiple leiomyomas. Cut surfaces were generally cream-white or cream-yellow, solid, and nodular.

FH immunohistochemical expression was lost in 17 of 19 tumors and retained in two. One FH-retained tumor harbored a germline *FH* variant of uncertain significance, p.(Gly364Arg) (Case 16), whereas germline testing was not performed in the other. Diffuse 2SC positivity was present in all cases. Individual tumor and immunohistochemical characteristics are shown in Table 2A.

Case 7 differed from the other cases, with a multilobulated, solid, pink, fleshy, and non-infiltrative gross appearance. Because leiomyosarcoma could not be excluded on preoperative MRI, both STUMP and leiomyosarcoma were considered in the differential diagnosis, and the tumor underwent additional histopathologic and immunohistochemical evaluation. Histopathologic examination showed no tumor cell necrosis, significant cytologic atypia, pleomorphism, bizarre nuclei, increased mitotic activity, or atypical mitotic figures. h-Caldesmon was positive, supporting smooth muscle differentiation, whereas HMB45 was negative, arguing against PEComa. PHH3 showed no immunoreactivity, and Ki-67 expression was approximately 3% in the hotspot, providing additional support for low proliferative activity. Taken together with FH loss and diffuse 2SC positivity, the overall morphologic and immunohistochemical findings favored a diagnosis of FH-deficient uterine leiomyoma rather than STUMP or leiomyosarcoma. No germline pathogenic/likely pathogenic *FH* variant was identified.

### 3.3. Genetic Results

**Figure 1:**
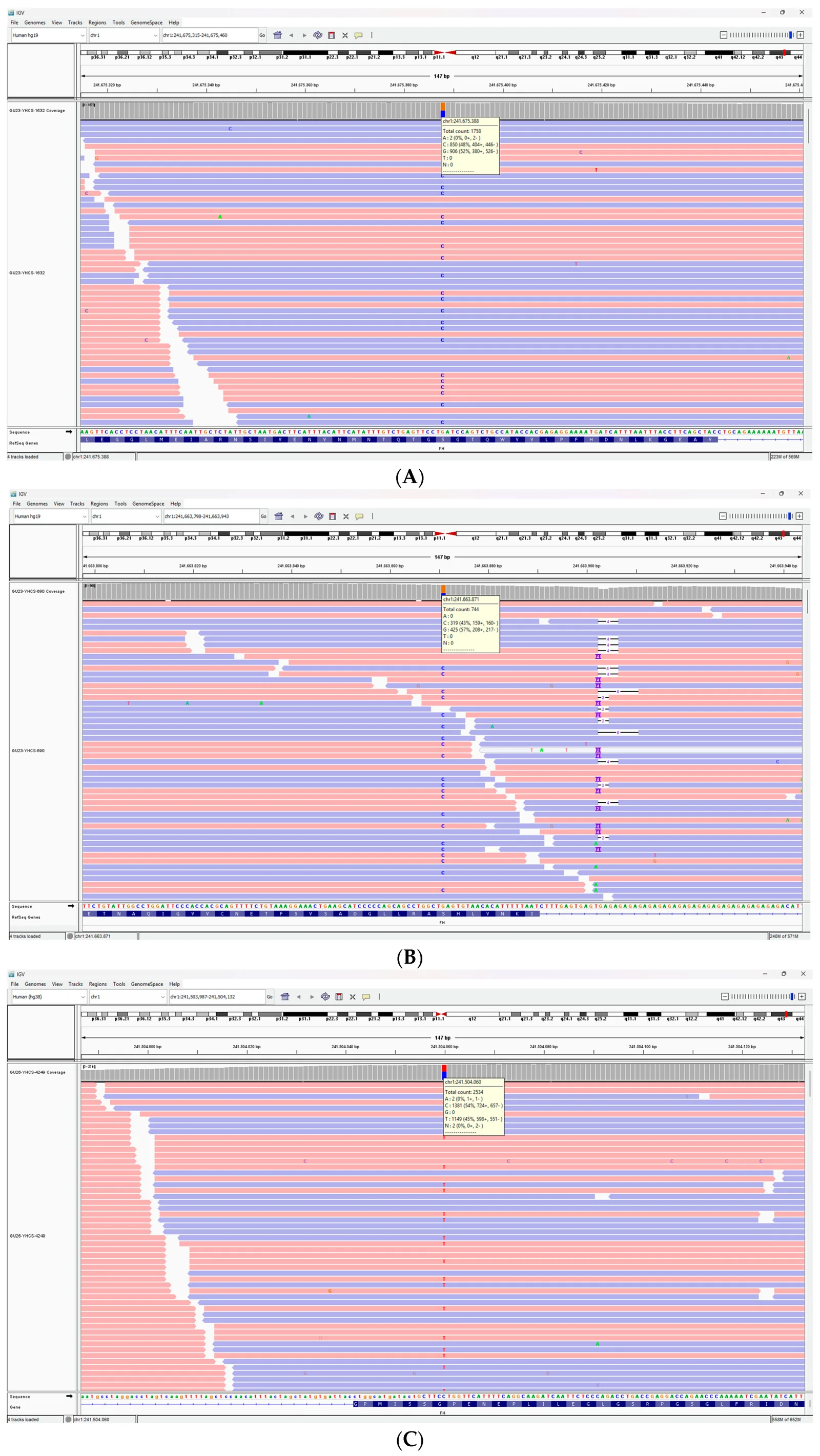
Representative IGV views of germline *FH* variants identified in the study cohort. (**A**) Pathogenic FH NM_000143.4.434C>G [p.(Ser145Ter)]; (**B**) likely pathogenic FH NM_000143.4.1256C>G [p.(Ser419Ter)]; and (**C**) FH NM_000143.4.1090G>A [p.(Gly364Arg)], classified as a variant of uncertain significance. All three variants were supported by adequate sequencing depth and allele distributions consistent with heterozygous germline variants. The colored bases and read alignments represent the standard IGV display of sequence reads and nucleotide substitutions.

Germline *FH* analysis results were available for 17 of the 19 patients. Pathogenic/likely pathogenic (P/LP) germline *FH* variants were identified in 2 of the 17 tested patients (2/17, 11.8%; 10.5% of the overall cohort). The *FH* NM_000143.4:c.434C>G [p.(Ser145Ter)] variant was classified as pathogenic (Case 10), whereas the *FH* NM_000143.4:c.1256C>G [p.(Ser419Ter)] variant was classified as likely pathogenic (Case 4). One additional patient harbored the *FH* NM_000143.4:c.1090G>A [p.(Gly364Arg)] variant, which was classified as a variant of uncertain significance (VUS) (Case 16; 1/17, 5.9%; 5.3% of the overall cohort). No pathogenic, likely pathogenic, or VUS germline *FH* variants were identified in the remaining 14 tested patients. Copy-number variant analysis using MUSKAT^®^ was performed in all 17 genetically tested patients, and no pathogenic or likely pathogenic *FH* copy-number variants were identified. Germline genetic testing could not be completed in two patients because they declined genetic evaluation (Cases 3 and 19) (Table 2B, Figure 1).

Both p.(Ser145Ter) and p.(Ser419Ter) were nonsense variants predicted to introduce premature termination codons and result in a loss of function. The p.(Ser145Ter) variant was classified as pathogenic according to the ACMG/AMP criteria based on its predicted loss-of-function effect, rarity in population databases, and previous pathogenic classification.

The p.(Ser419Ter) variant was classified as likely pathogenic because it is a nonsense alteration introducing a premature termination codon, loss of function is an established disease mechanism for *FH*-related disorders, the variant is absent from large population databases, and its high CADD PHRED score of 45 provides additional computational support for pathogenicity.

The p.(Gly364Arg) variant was a missense alteration located adjacent to the catalytic active site of the FH protein. The variant had a CADD PHRED score of 26.8, supporting a potentially deleterious effect, and pathogenic missense variants affecting adjacent residues within the same functional region have been reported. However, available clinical, segregation, and functional evidence remained insufficient to establish pathogenicity, and the variant was therefore classified as a VUS.

All three variants demonstrated adequate sequencing quality and coverage. The c.434C>G, c.1256C>G, and c.1090G>A variants were detected at read depths of 1,758×, 744×, and 2,450×, respectively, with corresponding variant allele fractions of 48%, 42%, and 46%, consistent with heterozygous germline variants.

### 3.4. Preoperative Imaging and Renal Evaluation Findings

Preoperative pelvic MRI was available in 12 patients. Among those with serial imaging, leiomyoma diameter increased by 11.1–150% over six months. The patient with the pathogenic p.(Ser145Ter) variant had no preoperative MRI because a 1.5 cm leiomyoma was incidentally identified and removed during cesarean delivery. The patient with the likely pathogenic p.(Ser419Ter) variant had a large leiomyoma without serial imaging, whereas the p.(Gly364Arg) VUS carrier showed a 17.1% increase over six months. No suspicious pelvic MRI findings were observed in either the likely pathogenic or VUS carrier.

In Case 7, preoperative MRI showed a heterogeneous, partially cystic mass with numerous vascular structures and marked diffusion restriction in the solid components. The radiology report described interval growth together with prominent vascularity and diffusion restriction; therefore, malignancy could not be excluded preoperatively. No germline pathogenic/likely pathogenic *FH* variant was identified, and final histopathology confirmed an FH-deficient uterine leiomyoma. Necrosis was reported in only one patient (Case 12), who also had no germline pathogenic/likely pathogenic *FH* variant and no additional suspicious findings on preoperative MRI.

Postoperative renal evaluation within the preceding 12 months was completed in 17 of 19 patients by ultrasonography, computed tomography, or magnetic resonance imaging. Pelvicalyceal dilatation was identified in two patients (Cases 3 and 5), while a parapelvic cyst was detected in one patient (Case 12). Germline *FH* testing had not been completed in one of the patients with pelvicalyceal dilatation (Case 3). Among the two patients without germline genetic testing, renal imaging had also not been completed in Case 19; therefore, hereditary risk assessment could not be fully completed in these patients. Renal CT was normal in the p.(Ser145Ter) carrier and renal MRI in the p.(Ser419Ter) carrier; neither had a renal mass or malignancy, and both were enrolled in annual renal imaging surveillance. No additional pathologic findings were identified on the remaining renal imaging studies.

### 3.5. Follow-Up and Recurrence

During postoperative follow-up, leiomyoma recurrence occurred in three of nine patients who underwent myomectomy. Two had no germline pathogenic/likely pathogenic FH variant, whereas one carried the likely pathogenic p.(Ser419Ter) variant. This patient was nulligravida at myomectomy, subsequently conceived, and three recurrent uterine leiomyomas of approximately 2 cm were detected at 8 weeks of gestation during routine obstetric ultrasonography. No recurrence occurred in the patient with the pathogenic p.(Ser145Ter) variant. Recurrence assessment was not applicable to the 10 patients who underwent hysterectomy.

Cascade genetic evaluation was recommended for the first-degree relatives of the two patients carrying germline P/LP *FH* variants; however, no family members presented for genetic evaluation during the follow-up period. Dermatologic evaluation was available for all patients, and no cutaneous leiomyomas were identified.

## 4. Discussion

We evaluated the clinical, histopathologic, immunohistochemical, germline genetic, renal, dermatologic, surgical, and follow-up characteristics of 19 patients with FH-deficient uterine leiomyomas. Among 17 patients who completed peripheral-blood germline *FH* testing, two had pathogenic/likely pathogenic variants and one had a variant of uncertain significance (VUS). Notably, the pathogenic p.(Ser145Ter) variant was identified after incidental detection of a solitary 1.5 cm leiomyoma during cesarean delivery. Conversely, Case 7 had MRI findings suspicious for malignancy and a tumor with FH loss and diffuse 2SC positivity, but no germline pathogenic/likely pathogenic *FH* variant. The patient with the likely pathogenic p.(Ser419Ter) variant conceived after myomectomy and developed three new leiomyomas during obstetric follow-up, illustrating that fertility may be preserved after myomectomy despite an ongoing risk of recurrence. Finally, the absence of cutaneous leiomyomas in our cohort underscores that germline *FH* predisposition cannot be excluded solely on the basis of absent cutaneous manifestations.

The frequency of germline *FH* variants in FH-deficient uterine leiomyomas varies widely across studies. Earlier estimates suggested a prevalence of approximately 2.7–13.9% [17], whereas higher rates have been reported in selected cohorts: 23.1% in Harrison et al. (3/13), 18.2% in Äyräväinen et al. (2/11), 22.0% in Kipnis et al. (9/41), and 34.1% in McHenry et al. (15/44) [18,19,20,21]. In the largest molecularly characterized series to date, Liu et al. reported germline *FH* mutations in 74 of 207 evaluable cases (35.7%) [22]. In our cohort, pathogenic/likely pathogenic variants were identified in 2 of 17 patients tested (11.8%), toward the lower end of the published range. This heterogeneity may reflect differences in cohort size, patient selection, diagnostic criteria, molecular methods, and geographic and genetic background.

Combined interpretation of FH and 2SC immunohistochemistry is important for diagnosing FH-deficient uterine leiomyomas. Joseph et al. reported 2SC positivity in all five tumors with *FH* abnormalities, whereas FH loss was present in only two; FH expression was retained in three tumors with missense variants [23]. Similarly, Kipnis et al. found FH loss in 11 of 20 cases and 2SC positivity in 16, including one patient with a pathogenic germline missense *FH* variant who retained FH expression despite positive 2SC staining [20]. Liu et al. also noted that, beyond the classic FH-negative/2SC-positive pattern, retained or weak/focal FH expression may occur, with 2SC providing complementary diagnostic value [22]. Furthermore, Chan et al. reported that, among six patients with HLRCC and confirmed germline *FH* mutations, FH expression was retained in all examined sections in two patients, whereas three patients showed variable FH staining across different leiomyomas, and emphasized that retained FH expression should not be used to exclude the possibility of HLRCC [24]. In our cohort, diffuse 2SC positivity was also observed in both cases with retained FH expression. In one of these cases, a germline p.(Gly364Arg) VUS located adjacent to the catalytic active site of the FH protein was identified. The presence of pathogenic missense variants affecting neighboring residues within the same functional region, together with supportive in silico evidence for the p.(Gly364Arg) variant, suggested a potentially deleterious effect. This finding underscores the need to avoid overlooking diffuse 2SC positivity in cases with retained FH expression and supports germline genetic evaluation and, when appropriate, referral for genetic counseling. Taken together with the available literature, our findings reinforce the importance of interpreting FH and 2SC immunohistochemistry in combination in morphologically suspicious cases.

Rogozhina et al. recently identified eosinophilic globules, staghorn-like vessels, and diffuse nuclear atypia as reproducible morphologic features with diagnostic value for FH deficiency [25]. However, clinical and morphologic features of FH-deficient uterine leiomyomas may suggest germline *FH* status but cannot reliably distinguish carriers from noncarriers. Shi et al. identified pathogenic germline *FH* variants in 8 of 12 tested patients; although characteristic histomorphologic features were more frequent in carriers, age and tumor size did not differ, and no single morphologic feature reliably predicted germline status [26]. McHenry et al. reported a younger median age in P/LP carriers than noncarriers (33 vs. 44 years) [21], while Liu et al. found median ages of 33 and 39 years in hereditary and sporadic cases, respectively, with substantial phenotypic overlap [22]. In our cohort, the two P/LP carriers were diagnosed at 35 and 34 years of age, respectively. Because there were only two P/LP carriers, this observation should be regarded as descriptive and hypothesis-generating only. Moreover, Case 7 lacked a germline P/LP variant despite a 15 cm radiologically suspicious tumor, whereas p.(Ser145Ter) was identified in an incidentally detected solitary 1.5 cm leiomyoma. Thus, age, tumor size, morphology, and imaging findings are insufficient to reliably predict germline *FH* predisposition.

Although FH deficiency is most commonly encountered in benign uterine leiomyomas, atypical histomorphologic features may complicate the differential diagnosis with smooth muscle tumors of uncertain malignant potential (STUMP) and uterine leiomyosarcoma. Pors et al. identified FH deficiency in 3 of 48 tumors (6.3%) previously classified as STUMP, and reported that these tumors exhibited features consistent with FH-deficient leiomyomas and followed a benign clinical course [27]. In contrast, Chapel et al. detected FH deficiency in 7 of 348 uterine leiomyosarcomas (2%), demonstrating that FH loss alone does not exclude malignancy [28]. In a 2025 review, D’Indinosante et al. emphasized that the detection of FH deficiency in uterine leiomyosarcoma does not directly establish malignant transformation from a pre-existing FH-deficient leiomyoma. They further noted that FH inactivation in uterine leiomyosarcoma may represent part of more complex genomic events associated with tumor genomic instability, including deletions and loss of heterozygosity, and that direct evidence supporting the malignant transformation of FH-deficient leiomyomas into leiomyosarcoma remains lacking [6]. In our cohort, leiomyosarcoma could not be excluded preoperatively in Case 7 because of prominent vascularity, marked diffusion restriction within the solid components, and intraperitoneal free fluid on MRI; on pathologic examination, the 15 cm tumor was grossly solid, pink-fleshy, and multilobulated. Histopathologic and immunohistochemical evaluation showed no increased mitotic activity or other findings supporting a malignant smooth muscle tumor (h-caldesmon positive, HMB45 negative, and no PHH3 immunoreactivity), supporting a diagnosis of FH-deficient leiomyoma. The absence of recurrence or evidence of metastatic disease during follow-up was also consistent with a benign clinical course. This case illustrates the diagnostic challenge created by overlapping benign and malignant features in FH-deficient uterine smooth muscle tumors and underscores the importance of integrating clinical, radiologic, gross, histomorphologic, and immunohistochemical findings for accurate classification.

FH-deficient uterine leiomyomas may require surgery at a relatively young age and may recur, which is particularly relevant in patients desiring fertility preservation. Liu et al. reported higher rates of nulliparity (54.1% vs. 26.4%) and previous myomectomy (44.6% vs. 11.5%) in hereditary than sporadic FH-deficient leiomyomas [22]. In our cohort, nine patients underwent myomectomy, including all five who were nulligravida at baseline and all three patients with a germline P/LP variant or VUS. The p.(Ser145Ter) and p.(Gly364Arg) carriers had previously given birth, whereas the nulligravida p.(Ser419Ter) carrier conceived approximately 41 months after myomectomy. No leiomyomas were detected at the gynecologic ultrasonographic examination performed one year after surgery or on the most recent available pre-pregnancy imaging. However, at 8 weeks of the current pregnancy, routine obstetric ultrasonography identified three new uterine leiomyomas measuring approximately 2 cm. The patient is currently being followed at 29 weeks of gestation, and serial ultrasonographic assessments have shown no significant growth of the leiomyomas; no medical or surgical intervention has been required. Current recommendations for FH tumor predisposition syndrome support the management of uterine leiomyomas according to symptomatic lesions and reproductive counseling needs [11]. Accordingly, given the small size of the lesions, absence of symptoms, stability during pregnancy, and the patient’s desire for future fertility, a conservative approach with close gynecologic surveillance was adopted. Vaginal delivery is planned in the absence of an obstetric contraindication, and close postpartum follow-up of the leiomyomas will be continued, with surgical management reconsidered if symptoms, significant growth, or other concerning features develop. Renal and dermatologic surveillance will also be continued because of the germline P/LP FH variant. Consistent with the recurrence tendency reported in the literature, this case illustrates that, although fertility may be preserved after myomectomy in FH-related uterine disease, new leiomyomas may still develop and the risk of recurrence may persist.

FH tumor predisposition syndrome encompasses a broad clinical spectrum, with cutaneous and uterine leiomyomas among its most common manifestations. Cutaneous leiomyomas occur in approximately 50–80% of carriers, uterine leiomyomas in 40–90% of affected women, and renal tumors in 10–15% [11]. Although less frequent, renal cell carcinoma is clinically critical because of its aggressive biologic behavior and its potential for early metastasis even from small primary tumors [11,29]. In the cohort of 185 individuals reported by Forde et al., renal tumors occurred in 12.4%, with an estimated lifetime risk of renal cell carcinoma of approximately 21% [29]; accordingly, regular renal imaging surveillance is recommended for carriers of germline P/LP FH variants [11,29]. No cutaneous leiomyomas were identified on dermatologic evaluation, and baseline renal imaging showed no renal mass or malignancy in either of the two P/LP variant carriers; both remain under annual surveillance. Overall, our findings indicate that the absence of cutaneous leiomyomas or renal tumors does not exclude germline FH predisposition and that an FH-deficient uterine leiomyoma may provide an opportunity for diagnosis before other syndromic manifestations emerge.

Family history may increase clinical suspicion: in our cohort, the only patient with a family history of renal cancer carried the pathogenic germline FH p.(Ser145Ter) variant and also had a family history of uterine leiomyoma. The coexistence of renal cancer and uterine leiomyoma within the family should therefore heighten suspicion for FH tumor predisposition syndrome and prompt a consideration of germline *FH* testing. However, a negative or unknown family history does not exclude the syndrome [11]. In our cohort, both patients with germline P/LP *FH* variants were counseled regarding familial risk and the importance of cascade genetic evaluation in first-degree relatives, and genetic testing for the familial *FH* variant was recommended for these relatives. In addition, the patient harboring the p.(Gly364Arg) VUS was referred to the Medical Genetics unit so that the potential clinical implications of the finding, including the supportive in silico evidence for a deleterious effect, could be explained in detail and appropriate genetic counseling could be provided. During follow-up, cascade genetic evaluation could not be completed in the first-degree relatives of the P/LP variant carriers. Identification of a familial *FH* variant in at-risk relatives is clinically important because the early recognition of carriers allows for the timely initiation of appropriate surveillance, particularly for renal malignancy, as well as dermatologic and gynecologic assessment. Current recommendations support annual renal MRI surveillance beginning at 10 years of age in carriers of pathogenic *FH* variants, complete dermatologic examination every 1–2 years from the time of diagnosis, and gynecologic assessment as clinically indicated for symptomatic lesions and reproductive counseling needs [11]. Thus, recognition of an FH-deficient uterine leiomyoma may have important implications, not only for the index patient but also for familial risk assessment and surveillance.

The main strength of this study is its integrated evaluation of clinical, histopathologic, immunohistochemical, and germline genetic features alongside renal imaging, dermatologic assessment, surgical management, fertility-related outcomes, and recurrence. Reflecting five years of experience at a tertiary referral center, the cohort contributes to a field in which reported germline *FH* variant frequencies remain heterogeneous and clinical-genetic data from Türkiye are limited. Genetic counseling and recommendations for cascade testing extended hereditary risk assessment beyond the index patients. Postoperative recurrence data and fertility-related outcomes after uterus-preserving surgery further enhance the study’s clinical relevance.

This study has several limitations. First, its single-center ambispective design and small sample size may limit the generalizability of the findings. Second, because somatic *FH* analysis of tumor tissue was not performed, patients without germline P/LP *FH* variants could not be classified as sporadic, and potential somatic *FH* alterations could not be assessed. Without paired tumor analysis, the direct contribution of a germline *FH* variant to the development of the corresponding leiomyoma cannot be fully determined. Future studies incorporating tissue-based next-generation sequencing together with CNV or MLPA analysis are needed to characterize second-hit somatic events, structural alterations, and biallelic *FH* inactivation. Histopathologic slides were not centrally re-reviewed, morphologic features were not reassessed using a standardized scoring system, and immunohistochemical data were obtained from the original pathology reports. In cases with retained FH expression, detailed staining patterns such as weak, focal, mosaic, or partial expression were not documented in the original reports; therefore, further subclassification of retained FH expression was not possible. Germline genetic testing was incomplete in two patients, and renal imaging was incomplete in two. In addition, although cascade genetic evaluation was recommended for the first-degree relatives of patients carrying germline P/LP *FH* variants, no family members presented for genetic evaluation; therefore, carrier status among relatives could not be determined, and familial genetic assessment could not be completed. Because follow-up duration varied among patients and the cohort was small, recurrence, fertility, and renal outcomes should be interpreted descriptively. Finally, the sample size precludes definitive conclusions regarding genotype–phenotype associations or the clinical effects of specific germline *FH* variants.

## 5. Conclusions

FH-deficient uterine leiomyomas may indicate underlying germline *FH* predisposition. Patients with pathologically identified FH deficiency should undergo genetic counseling, germline evaluation, and integrated follow-up addressing renal surveillance, dermatologic assessment, fertility-related considerations, and leiomyoma recurrence. Obstetricians and gynecologists play a key role in initiating and coordinating post-surgical genetic evaluation and syndrome-specific surveillance. Larger multicenter studies incorporating paired tumor and germline analyses are needed to better define molecular features and genotype–phenotype associations.

## Figures and Tables

**Table 1:** Demographic, clinical, and surgical characteristics of the study cohort.

Characteristic	Overall Cohort (*n* = 19)
Age, years, mean ± SD	44.9 ± 7.2
Age range, years	34–60
Body mass index, kg/m^2^, mean ± SD	28.6 ± 6.5
Menopausal status	
Premenopausal	9 (47.4%)
Postmenopausal	10 (52.6%)
Gravidity, median (IQR)	3.0 (2.5)
Parity, median (IQR)	2.0 (1.0)
Nulligravida	5 (26.3%)
Largest leiomyoma diameter, cm, median (IQR)	6.5 (4.5–8.0)
Age at first leiomyoma diagnosis, years, median (IQR), *n* = 14	35.5 (30.3–40.0)
Previous leiomyoma surgery	
Yes	3 (15.8%)
No	16 (84.2%)
Clinical presentation	
Abnormal uterine bleeding	8 (42.1%)
Pelvic pain	4 (21.1%)
Abnormal uterine bleeding and pelvic pain	3 (15.8%)
Asymptomatic	4 (21.1%)
Family history of uterine leiomyoma	
Yes	11 (57.9%)
No	6 (31.6%)
Unknown	2 (10.5%)
Family history of renal cancer	
Yes	1 (5.3%)
No	16 (84.2%)
Unknown	2 (10.5%)
Surgical procedure	
Hysterectomy	10 (52.6%)
Abdominal myomectomy	9 (47.4%)
Follow-up duration, months, median (IQR)	30 (16–37)

Data are presented as n (%) unless otherwise indicated. Age at first leiomyoma diagnosis was available for 14 patients. Largest leiomyoma diameter was defined as the greatest dimension recorded on pathological examination. Abbreviations: BMI, body mass index; IQR, interquartile range; SD, standard deviation.

**Table 2:** Individual leiomyoma-related, imaging, immunohistochemical, germline genetic, and follow-up characteristics of patients with FH-deficient uterine leiomyomas. (**A**) Individual clinical, imaging, and immunohistochemical characteristics of patients with FH-deficient uterine leiomyomas. (**B**) Individual germline genetic, renal surveillance, dermatologic, and follow-up characteristics of patients with FH-deficient uterine leiomyomas.

(**A**)									
**Case**	**Age**	**Family History of Leiomyoma †**	**Family History of Renal Cancer †**	Largest Leiomyoma Diameter (cm) *	Leiomyoma Number **	Leiomyoma Growth Within 6 Months Preoperatively ***	**Preoperative MRI Findings**	**FH IHC**	**2SC IHC**
1	51	Yes	No	6.5	Single	Unknown	Not performed	Loss	DP
2	41	No	No	3.5	Single	Unknown	Not performed	Loss	DP
3	60	No	No	2.5	Single	Unknown	No suspicious findings	Retained	DP
4	34	Unknown	Unknown	11	Multiple	Unknown	No suspicious findings	Loss	DP
5	54	Yes	No	4.5	Single	18.2% increase	No suspicious findings	Loss	DP
6	54	Yes	No	8	Multiple	150.0% increase	Not performed	Loss	DP
7	41	Unknown	Unknown	15	Single	Unknown	Suspicious MRI features; malignancy not excluded	Loss	DP
8	43	No	No	5	Multiple	11.1% increase	No suspicious findings	Loss	DP
9	46	Yes	No	6	Single	Unknown	Not performed	Loss	DP
10	35	Yes	Yes	1.5	Single	Unknown	Not performed	Loss	DP
11	45	Yes	No	7.5	Single	64.3% increase	No suspicious findings	Loss	DP
12	38	Yes	No	4	Single	Unknown	Necrosis present	Loss	DP
13	53	Yes	No	5	Multiple	45.3% increase	Not performed	Loss	DP
14	47	No	No	7	Multiple	Unknown	No suspicious findings	Loss	DP
15	41	Yes	No	5	Multiple	27.7% increase	No suspicious findings	Loss	DP
16	36	Yes	No	8	Multiple	17.1% increase	No suspicious findings	Retained	DP
17	48	No	No	12	Multiple	Unknown	No suspicious findings	Loss	DP
18	40	Yes	No	10	Single	50.0% increase	No suspicious findings	Loss	DP
19	46	No	No	8	Single	100.0% increase	Not performed	Loss	DP
(**B**)									
**Case**		**Germline FH Result**		**Renal Imaging Findings**		**Cutaneous Leiomyoma**	**Follow-Up, Months**	**Leiomyoma Recurrence**	
1		No P/LP/VUS detected		CT, normal		Absent	60	No	
2		No P/LP/VUS detected		US, normal		Absent	51	Yes	
3		Not tested		CT, pelvicalyceal dilatation		Absent	47	Not applicable	
4		Likely pathogenic (p.Ser419Ter)		MRI, normal		Absent	41	Yes	
5		No P/LP/VUS detected		CT, pelvicalyceal dilatation		Absent	36	Not applicable	
6		No P/LP/VUS detected		CT, normal		Absent	14	Not applicable	
7		No P/LP/VUS detected		MRI, normal		Absent	8	No	
8		No P/LP/VUS detected		MRI, normal		Absent	24	Yes	
9		No P/LP/VUS detected		US, normal		Absent	38	Not applicable	
10		Pathogenic (p.Ser145Ter)		CT, normal		Absent	24	No	
11		No P/LP/VUS detected		MRI, normal		Absent	26	Not applicable	
12		No P/LP/VUS detected		US, parapelvic cyst		Absent	33	No	
13		No P/LP/VUS detected		CT, normal		Absent	33	Not applicable	
14		No P/LP/VUS detected		MRI, normal		Absent	30	Not applicable	
15		No P/LP/VUS detected		MRI, normal		Absent	30	Not applicable	
16		VUS (p.Gly364Arg)		MRI, normal		Absent	18	Not applicable	
17		No P/LP/VUS detected		Not completed		Absent	12	Not applicable	
18		No P/LP/VUS detected		MRI, normal		Absent	9	No	
19		Not tested		Not completed		Absent	3	No	

(**A**) Abbreviations: DP, diffuse positive; FH, fumarate hydratase; IHC, immunohistochemistry; MRI, magnetic resonance imaging; 2SC, S-(2-succino)cysteine. † “Unknown” in the family history columns indicates that the patient was unable to provide reliable information regarding the relevant family history. * Largest leiomyoma diameter was defined as the greatest dimension recorded on pathological examination. ** Leiomyoma number was determined based on pathological examination of the surgical specimen. *** Percentage change in leiomyoma size was calculated using the largest diameter reported on serial preoperative imaging according to the following formula: [(final diameter − initial diameter)/initial diameter] × 100. When serial preoperative imaging was unavailable, leiomyoma growth was recorded as “Unknown.” (**B**) Abbreviations: CT, computed tomography; FH, fumarate hydratase; MRI, magnetic resonance imaging; P/LP, pathogenic/likely pathogenic; US, ultrasonography; VUS, variant of uncertain significance.

## Data Availability

The data supporting the findings of this study are available from the corresponding author upon reasonable request, subject to institutional and ethical restrictions.

## Abbreviations

2SC	S-(2-succino) cysteine
ACMG/AMP	American College of Medical Genetics and Genomics/Association for Molecular Pathology
BMI	Body mass index
CNV	Copy-number variant
CT	Computed tomography
FH	Fumarate hydratase
HLRCC	Hereditary leiomyomatosis and renal cell cancer
IHC	Immunohistochemistry
IQR	Interquartile range
MLPA	Multiplex ligation-dependent probe amplification
MRI	Magnetic resonance imaging
NGS	Next-generation sequencing
P/LP	Pathogenic/likely pathogenic
RCC	Renal cell carcinoma
VUS	Variant of uncertain significance
